# Effect of a 14-day course of systemic corticosteroids on the hypothalamic-pituitary-adrenal-axis in patients with acute exacerbation of chronic obstructive pulmonary disease

**DOI:** 10.1186/1471-2466-8-1

**Published:** 2008-01-26

**Authors:** Philipp Schuetz, Mirjam Christ-Crain, Ursula Schild, Erika Süess, Michael Facompre, Florent Baty, Charly Nusbaumer, Martin Brutsche, Beat Müller

**Affiliations:** 1 Division of Endocrinology, Diabetes and clinical Nutrition, University Hospital Basel, Petersgraben 4, CH-4031 Basel. Switzerland; 2 Division of Pneumology, Department of Chemical Pathology, University Hospital Basel, Petersgraben 4, CH-4031 Basel. Switzerland; 3 Department of Internal Medicine, University Hospital Basel, Petersgraben 4, CH-4031 Basel. Switzerland

## Abstract

**Background:**

As supra-physiological intake of corticosteroids is a well known risk factor for the development of adrenal insufficiency, we investigated the function of the hypothalamic-pituitary-adrenal (HPA) axis during a 14-day course of systemic corticosteroids in patients with acute exacerbation of chronic obstructive pulmonary disease using clinical and laboratory measures.

**Methods:**

A systematic clinical and laboratory assessment including measurement of basal cortisol levels and the response to low dose (1 μg) ACTH stimulation was performed in nine patients before, on the first and the last day of treatment, as well as 2, 7 and 21 days after corticosteroid withdrawal.

**Results:**

At baseline, all nine patients had normal responses to 1 μg ACTH. On the first day of steroid treatment, 78% had a blunted peak cortisol response. This percentage increased to 89% after 14 days of steroid treatment. 78%, 33% and 33% of the patients had a blunted cortisol response to ACTH 2, 7, and 21 days after corticosteroid withdrawal, respectively. ROC curve analysis revealed that only basal cortisol concentrations (AUC 0.89), but not ACTH concentrations (AUC 0.49) or clinical signs (AUC 0.47) were predictive of an impaired function of the HPA axis. Basal cortisol levels of > 400 and < 150 nmol/l were 96% and 100% sensitive for a normal or pathological response to the ACTH stimulation test, respectively.

**Conclusion:**

Immediate and prolonged suppression of the HPA axis is a common finding in otherwise asymptomatic patients undergoing systemic steroid treatment for acute exacerbation of chronic obstructive pulmonary disease and can reliably be assessed with the low-dose ACTH test.

## Background

The administration of a 2 week course of systemic corticosteroids to treat patients with acute exacerbations of chronic obstructive pulmonary disease (AECOPD) has become common practice over the past 30 years [1]. This practice, however, remains debated because corticosteroids are only modestly effective in shortening the duration of exacerbations of COPD and have serious metabolic adverse effects including hyperglycemia, osteoporosis, myopathies, mental disturbances and suppression of the hypothalamic-pituitary-adrenal (HPA) axis, among others [2].

Adrenal insufficiency often presents with only minimal and unspecific clinical prodromal symptoms but may suddenly become life-threatening if left untreated upon acute stress (e.g. infection, acute exacerbation, trauma or critical illness)[3]. Predicting adrenal insufficiency is challenging, since neither the dose nor the duration of glucocorticoid treatment, nor random plasma cortisol measurements correlate with the function of the HPA axis [4]. The insulin-induced hypoglycaemia test is the most agreed-on reference standard for "stress" and for the verification of adrenal insufficiency [5]. The insulin tolerance test, however, is labour-intensive, potentially dangerous and unpleasant for the patient, and thus, not feasible outside an endocrine unit. In clinical practice, the simpler low-dose corticotropin (ACTH) test is a useful and sensitive substitute as it reveals partial adrenal insufficiency by providing physiological adrenocortical stimulation [6,7].

The aim of this study was to assess occurrence and time course of adrenal suppression in patients undergoing a 14 day course of systemic corticosteroids for AECOPD by means of the low-dose corticotropin test.

## Methods

From October 2005 to June 2006, we prospectively included nine consecutive patients aged 40 years or older, who were admitted to the University Hospital in Basel, Switzerland, for acute exacerbation of COPD with >1 of the following symptoms: increase in dyspnea, increase in sputum, discolouration of sputum [8]. In all patients pneumonia was excluded by chest radiograph. All patients had a standardized steroid treatment with a single injection of 40 mg of methylprednisolone (Solumedrol^®^), followed by an oral 13-day regime of 40 mg prednisone (Prednisone^®^). In addition, in all patients a medical treatment with inhaled long acting β2-agonists and inhaled corticosteroids was initiated. Patients who received systemic glucocorticoid treatment within the last 6 months and patients with known Addison's disease, adrenal metastases, Cushing's disease or pituitary adenomas were excluded from the study.

After study inclusion, all patients were examined on 5 clinical visits, namely on days 2 and 14 (the second and the last day of steroid treatment) and on days 16, 21 and 35, respectively. On every visit, the patients' functional status and a clinical questionnaire, adapted from a published clinical adrenal insufficiency score were assessed (range 0–10 for each symptom, higher scores indicate greater discomfort) [9]. A subset of patients (n = 5) was asked at different time points to self-report their functional status with the help of a visual analogue scale, ranging from 0 (feeling extremely ill) to 100 (feeling completely healthy).

The study was approved by the institutional review board (Ethikkommission beider Basel, EKBB) and written informed consent was obtained from all included patients. All data were held and analyzed by the authors.

Prior to the initial administration of intravenous methylprednisolone and in the morning of all 5 clinical visits, low-dose (1 μg) ACTH tests were performed by a specialist study nurse. On all occasions, blood samples were taken at 0 min for the assessment of basal cortisol and basal ACTH concentrations, and again 30 min after intravenous (i.v.) administration of 1 μg corticotropin (synthetic ACTH_1–24_) for the measurement of stimulated serum cortisol concentrations. Based on prior studies in our Institution and the endocrine literature, a normal response to intravenous corticotropin was defined as a stimulated plasma cortisol concentration > 550 nmol/l [7,10-13]. Blood samples were immediately centrifuged, aliquoted, and stored at -70°C until batch analyses. Serum total cortisol was measured using a chemiluminescence immunoassay (DPC^® ^Diagnostic Products Corporation, Los Angeles, CA) with a calculated sensitivity of 13.8 nmol/l. The intra-assay coefficient of variation was 4.4%, and the inter-assay coefficient of variation was 11.0%. Serum ACTH was measured using a chemiluminescence immunoassay (DPC^® ^Diagnostic Products Corporation) with a sensitivity of 1 ng/l.

To evaluate differences between groups, the Mann-Whitney U test and the Wilcoxon signed rank sum test for not normally distributed unpaired and paired variables, and the Fisher's exact test for categorical variables were used, as appropriate. To compare the prognostic value of individual laboratory markers for predicting HPA insufficiency as assessed by the corticotropin test, a receiver operating characteristic (ROC) analysis was performed [14]. The area under the ROC curve (AUC) was the measure of the accuracy of the laboratory parameter to distinguish the groups. A *p*-value <0.05 (for a 2-sided test) was considered statistically significant. All calculations were performed using Stata 9.2 (Stata Corp, College Station, Tex).

## Results

### Baseline characteristics

We included nine steroid-naive patients (3 women, 6 men) with a median duration of COPD of 2 years (IQR 1–4) and a median of 1 (IQR 1–2) hospital admission for exacerbation per year. Seven patients were on medical treatment for COPD on admission, including inhaled short- and/or long acting β2-agonists and/or anticholinergics (78%) and inhaled corticosteroids (56%). None of the patients was admitted to the ICU or was in need of mechanical ventilation. The detailed baseline characteristics, clinical findings and laboratory data on admission are presented in Table 1.

**Table 1:** Baseline values for all patients (n = 9)

**Baseline characteristics**	
**Gender **(*female/male*)	3/6
**Age **(*years*)*	72 (51–86)
**Body-mass index **(*kg/m*^2^)*	25.9 (24.7–33.7)
**Smoking **(*pack years*)*	30 (25–50)
**Duration of COPD **(years)*	2.0 (1–4)
**Number of AECOPD **in previous year*	1 (1–2)
**Duration of AECOPD **(days)	4 (4–7)
**Dyspnea **(%)	9 (100%)
**Cough **(%)	9 (100%)
**Increased sputum production **(%)	9 (100%)
**Discoloured sputum **(%)	7 (78%)
**Fever **(%)	7 (78%)
**Maintenance therapy at admission **(%)	
β2-agonist and/or anticholinergic	7 (78%)
Inhaled corticosteroids	4 (44%)
Oral corticosteroids	0 (0%)
Theophyllin	0 (0%)
Long-term oxygen therapy	1 (11%)
**Severity of AECOPD – Anthonisen's criteria **[8] (%)	
1	0 (0%)
2	2 (22%)
3	7 (78%)

Heart rate* (beats/min)	84 (70–101)

Blood pressure* (mmHg)	
Systolic	143 (120–150)
Diastolic	80 (69–85)

Respiratory rate*	16 (12–25)

***Laboratory findings:***	

Leukocytes total*	9.3 (7.7–10.1)
**CRP **(mg/dl)*	19 (6–61)
**Cortisol basal **(nmol/l)**	439 (392–462)
**Cortisol after 1 μg ACTH **(nmol/l)**	712 (608–730)
**Basal ACTH **(ng/l)**	9.2 (5.1–11.5)

* median, interquartile range;

### Adrenal function test

**Figure 1:**
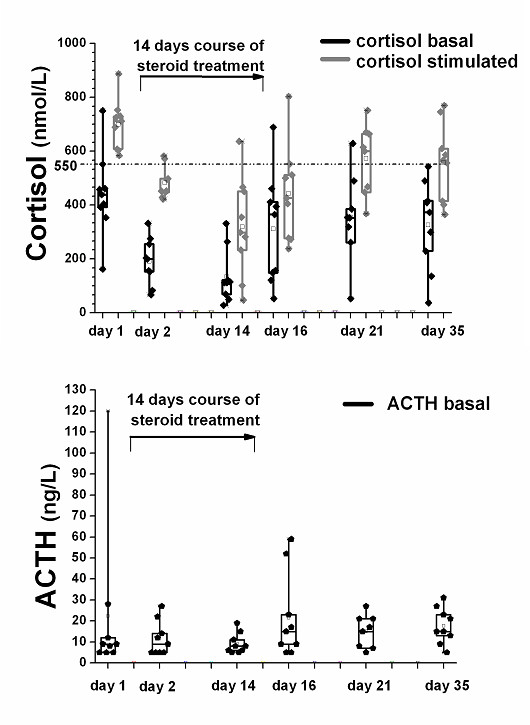
**(a) **Cortisol values basal (black) and after stimulation with 1 μg corticotropin (grey) and **(b) **corresponding basal ACTH concentrations for all patients at six clinical visits.

Figure 1 shows the basal and the stimulated cortisol concentrations and the corresponding basal ACTH values for all patients on baseline, before the initiation of steroid therapy, and in the morning of the five clinical visits. Compared to baseline (712 (95%CI 627–771)), median stimulated cortisol measurements were significantly lower on days 2, 14, 16 and 35 (453 (95%CI 438–529), p = 0.008; 298 (95%CI 177–461), p = 0.008; 427 (95%CI 307–579), p = 0.008 and 566 (95%CI 447–668), p = 0.04). In addition, median basal cortisol measurements 439 (95%CI 320–562)) were significantly lower on days 2 and 14 (200 (95%CI 124–259), p = 0.008 and 112 (95%CI 58–211), p = 0.007).

Before the initiation of steroid therapy, all patients (100%) had a normal rise of cortisol after injection of 1 μg ACTH defined as a stimulated cortisol value > 550 nmol/l [7,11,12]. On the first day of steroid treatment, 78% of patients had a blunted peak cortisol response. This percentage increased to 89% after 14 days of steroid treatment. 78%, 33% and 33% of the patients had a blunted cortisol response to 1 μg ACTH injection, 2, 5, and 14 days after corticosteroid withdrawal, respectively.

Median basal plasma cortisol (nmol/l) concentrations were significantly higher in patients with a normal adrenal response as compared to patients with a suppressed adrenal response (409 (95%CI 366–482) vs 142 (IQR 129–218)), *p *< 0.0001). Median ACTH concentrations (pmol/ml) were similar on baseline as compared to the 5 clinical visits (*p *> 0.05 for each measurement) and for all patients with normal and insufficient rise of cortisol after injection of 1 μg ACTH (14.4 (95%CI 11.3–21.7) vs 8.6 (95%CI 8.3–16.1), *p *= 0.1).

### Clinical data

**Figure 2:**
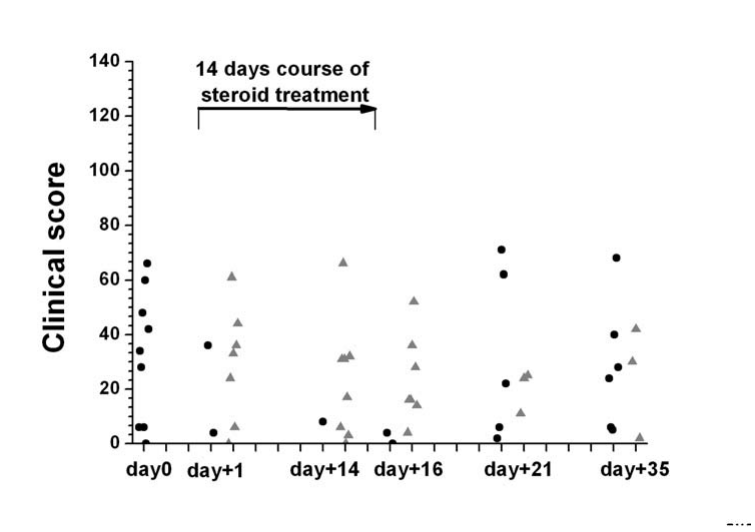
Clinical symptom score in patients with normal (●) and inadequate (▲) response to the 1 μg corticotropin test for each visit. The clinical score asks patients for fatigue, hypoglycemia (hypoglycemic symptoms or fasting glucose < 3,5 mmol), loss of energy, orthostatic disturbance or hypotonia (systolic blood pressure < 100 mmHg, diastolic blood pressure < 60 mmHg), reduced strength, sleep disturbance, muscle pain, mood changes (feeling depressed), nausea, concentration disturbance, weight loss (> 3 kg), stomach pain, hyperpigmentation and eosinophila (> 330 cells/ml) [9].

The symptom score suggestive of adrenal insufficiency was not discriminative for all 6 time points and among patients with normal and inadequate rise of cortisol in the ACTH test (Table 2 and Figure 2) [9]. The self-reported functional status assessed by the visual analogue scale on the different clinical visits did not significantly differ between clinical visits and time points of normal and pathological stimulated cortisol values, respectively (data not shown).

**Table 2:** Baseline cortisol concentration, cortisol concentration after stimulation with 1 μg corticotropin and clinical data of all 9 patients and all 6 clinical visits.

***Patient Nr*.**	***Laboratory and clinical parameters***	***Clinical visits***					
		***V0***	***V1***	***V2***	***V3***	***V4***	***V5***

**1**	Cortisol basal*	552	255	264	689	NA	543
	Cortisol stimulated*	887	572	467	803	NA	770
	Clinical score	6	4	0	0	NA	40

**2**	Cortisol basal*	162	153	112	53.2	53.1	36.8
	Cortisol stimulated*	603	582	355	277	368	401
	Clinical score	48	36	31	36	24	42

**3**	Cortisol basal*	439	156	69.2	149	261	416
	Cortisol stimulated*	753	448	101	238	448	566
	Clinical score	60	33	17	16	11	5

**4**	Cortisol basal*	404	66.6	121	157	352	373
	Cortisol stimulated*	608	422	451	405	671	587
	Clinical score	28	61	66	52	71	68

**5**	Cortisol basal*	392	200	112	461	385	409
	Cortisol stimulated*	584	453	298	553	600	610
	Clinical score	0	6	3	4	6	6

**6**	Cortisol basal*	462	82.6	50.1	365	319	299
	Cortisol stimulated*	726	429	233	427	468	557
	Clinical score	34	24	31	16	25	28

**7**	Cortisol basal*	750	332	27.0	121	629	229
	Cortisol stimulated*	730	496	46.5	273	752	415
	Clinical score	66	36	32	28	62	30

**8**	Cortisol basal*	458	204	115	411	354	136
	Cortisol stimulated*	689	452	282	501	615	365
	Clinical score	6	0	6	4	2	2

**9**	Cortisol basal*	353	275	331	394	490	490
	Cortisol stimulated*	712	498	637	512	664	746
	Clinical score	42	44	8	14	22	24

*(nmol/l)

### Prediction of adrenal insufficiency

**Figure 3:**
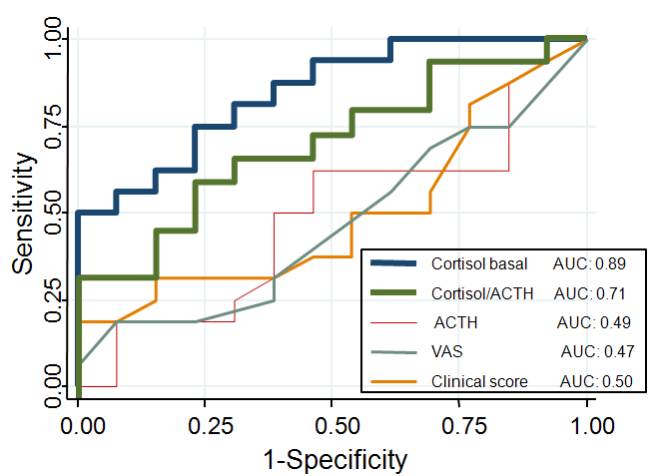
ROC curve analysis to predict adrenal insufficiency for basal cortisol, ACTH, the cortisol/ACTH ratio and the clinical score.

To compare the predictive value of different parameters (e.g. basal cortisol, basal ACTH, the cortisol/ACTH ratio, the clinical symptom score and the visual analogue scale) we pooled the data of all nine patients and visits, and performed ROC plot analysis (Figure 3). Sensitivity was calculated with those patients and time points, where the low dose corticotropin stimulation test was inadequate (n = 28) and specificity was assessed with those patients and time points, where it was normal (n = 25) (defined as rise of cortisol > 550 mmol/l after injection of 1 μg ACTH). The area under the ROC curve (AUC) to predict adrenal insufficiency revealed an AUC for basal cortisol of 0.91 (95%CI 0.85–0.99), which was better than for ACTH (0.63 (95%CI 0.48–0.79)), the ACTH/cortisol ratio (0.79 (95%CI 0.67–0.91)), the clinical symptoms score (0.51 (95%CI 0.34–0.68)) and the visual analogue scale (0.45 (95%CI 0.23–0.66)). Basal cortisol levels of > 400 and < 150 nmol/l were 96% and 100% predictive for a normal or pathological response to the ACTH stimulation test (Table 3). Serum ACTH levels and the ACTH/cortisol ratio did not improve the predictive accuracy of basal cortisol measurement alone.

**Table 3:** Sensitivity, specificity, percentage of correctly classified patients and positive and negative likelihood ratio of basal cortisol at different cut off values

***Cut-off value of basal cortisol***	**Sensitivity**	**Specificity**	***Correctly classified***	***LR+***	***LR-***
**<150 **nmol/l	100%	50%	74%	2.0	0.0
**<330 **nmol/l	80%	86%	83%	5.6	0.2
**>400 **nmol/l	56%	96%	77%	15.7	0.5

*LR*+ = positive likelihood ratio, *LR- *= negative likelihood ratio

## Discussion

Patients prescribed an oral and inhaled corticosteroid are at a dose related increased risk of adrenal crisis as illustrated in a recently published epidemiological case control study of 154 reported cases in the general population [15]. The magnitude of the risk for an individual patient with oral and inhaled corticosteroids for AECOPD, however, has not yet been determined and is often neglected in common practice [16]. A sensitive measure to assess the functional status of the HPA-axis, and thus the risk for Addisonian crises upon stress, is the low-dose ACTH-test. In this study, even short-term low-dose courses of systemic corticosteroids for the treatment of AECOPD in steroid naive patients was associated with a measurable and prolonged suppression of the HPA-axis in the majority of patients. Importantly, clinical signs and symptoms are unreliable and unable to predict an impairment of the HPA-axis. Accordingly, in patients with AECOPD receiving systemic corticosteroids the presence of adrenal insufficiency should be considered.

Physiologically, the hypothalamus secretes corticotropin releasing hormone (CRH), which stimulates the release of adrenocorticotropic hormone (ACTH) from the pituitary gland. ACTH leads to the release of cortisol through stimulation of the adrenal cortex, which in turn has a negative feedback on CRH and ACTH. Administration of exogenous corticosteroids, even in small doses for only few days, leads to a measurable suppression of the HPA-axis resulting in the inability of the adrenal cortex to secrete additional cortisol if needed [17]. One might argue that laboratory changes in the HPA axis after short-term systemic steroid therapy are not of clinical relevance. Indeed, the absolute risk of adrenal crisis after cessation of oral and inhaled corticosteroids might be considered rare based on the literature, but is likely to be substantially underreported in clinical practice [15]. Importantly, the manifestation and the severity of clinical signs depend on the presence of stress with its resulting increased cortisol demand. Upon major stress, even a mild adrenal insufficiency can be hazardous leading to hemodynamic instability, vasoactive-refractory shock and ultimatively death [3,15,18]. Consequently, in adrenal insufficiency administration of systemic corticosteroids (preferably hydrocortisone) is mandatory and potentially lifesaving. Unfortunately, symptoms of adrenal insufficiency are highly variable and only occur if the patient is experiencing more or less severe stress. Therefore, predicting or excluding the risk for adrenal insufficiency from clinical parameters, as often falsely done, is delicate and potentially dangerous. Illustratively, typical symptoms of adrenal insufficiency (e.g. weakness, low blood pressure and hypoglycemia) were completely nonspecific in this study and were, thus, unreliable predictors of adrenal insufficiency. This should be kept in mind before an absence of adrenal insufficiency is postulated based on the absence of signs and symptoms by the treating physician.

Based on our data and supported by the literature, basal cortisol levels are helpful to diagnose adrenal insufficiency in a subset of patients, i.e. if cortisol values are < 150 or > 400 nmol/l adrenal insufficiency is highly likely or can be excluded, respectively. Measurement of basal ACTH concentration did not improve the diagnostic accuracy. This is not surprising because of the pulsatile secretion of ACTH.

In asthmatic patients, the various adverse effects of oral and even inhaled corticosteroids on the HPA axis, including adrenal crisis, have been well recognized [18,19]. In patients receiving systemic corticosteroids for acute exacerbation of COPD, the frequency and duration of adrenal suppression has not prospectively been investigated. Surprisingly, in common practice, a so called "short-term" 14 days course of systemic corticosteroids is generally considered safe and the potential consequences of adrenal suppression are often neglected [16]. In our patients, adrenal stimulation tests revealed a suppressed adrenal response to corticotropin in 7 out of 9 patients after only one day of treatment and in 8 out of 9 patients after the 2 weeks course. Similar proportions have been reported from studies on longer-term glucocorticoid treatment [20]. The observed gradual recovery of the suppressed adrenal response to the low-dose corticotropin test was heterogeneous among the patients lasting from a few days to up to 3 weeks after withdrawal. In individual patients, it is not possible to predict the duration of the HPA axis recovery, though, careful instruction and observation of these patients and examination for potential adrenal insufficiency should be recommended.

This study has limitations. Firstly, we have only included a small, but well defined number of patients to assess the adrenal response after steroid treatment for AECOPD. The number of performed tests is too small to recommend definitive cut-off levels for peak cortisol concentrations during acute illnesses. Secondly, our population varied in respect to the use of inhaled corticosteroids on admission, which is a known risk factor for adrenal insufficiency. However, all patients had a normal ACTH stimulation test at baseline and the inhaled medical treatment with inhaled long acting β2-agonists and corticosteroids after study inclusion was uniform. Still, the impact of inhaled corticosteroids can not be addressed with this study. Thirdly, *we did not directly assess free cortisol values or albumin or cortisol binding globulin concentrations to calculate free cortisol levels*. Depending on the severity and kinetic of acute disease, total and free cortisol levels may show discordant results and total cortisol levels may not adequately anticipate the free cortisol levels needed during severe peracute stress [21].

## Conclusion

In conclusion, short-term, low-dose systemic steroid treatment for AECOPD exposes the patients to the risk of adrenal insufficiency. During the period of recovery the function of the adrenal response to stress may still be impaired. In a minority of patients the adrenal response even remained suppressed for several weeks. If patients are exposed to stress after such treatment, adrenal function should be assessed.

## Competing interests

The author(s) declare that they have no competing interests.

## Authors' contributions

MCC, MB, ES and BM had the idea for the study and directed the study design. PS, MCC, US, and ES directed the data collection and analysis and writing of the report. PS analyzed the data and wrote the first report. PS, MCC, US, ES, MF, FB, CN, MB and BM had substantial contributions in planning of the study, data collection, interpretation of data and/or writing of the manuscript.

## Pre-publication history

The pre-publication history for this paper can be accessed here:


